# Account of Diseases in an Eastern District of London, from the 20th of August to the 20th of September, 1800

**Published:** 1800-11

**Authors:** 


### STATE OF DISEASES IN LONDON.

*Account of Diſeaſes in an Eaſtern Diſtrict of London, from the 20th of Auguſt to the 20th of September, 1800.*

| | No. of Caſes. |
|---|---|
| ACUTE DISEASES. | |
| Typhus - - - - - - - | 7 |
| Dyſenteria - - - - - | 8 |
| Cholera Morbus - - - | 17 |
| CHRONIC DISEASES. | |
| Cough - - - - - - - | 12 |
| Cough with Dyſpnœa - - | 5 |
| Pertuſſis - - - - - - - | 2 |
| Hæmoptyſis - - - - - | 3 |
| Hoarſeneſs - - - - - - | 1 |
| Phthiſis Pulmonalis - - - | 4 |
| Catarrhus - - - - - - | 3 |
| Gaſtrodynia - - - - | 9 |
| Dyſpepſia - - - - - | 8 |
| Diarrhœa - - - - - - | 20 |
| Colica Pictonum - - - | 3 |
| Prolapſus Ani - - - - | 2 |
| Inteſtinal Hæmorrhagy - | 2 |
| Amenorrhœa - - - - - | 5 |
| Menorrhagia - - - - - - | 4 |
| Hemiplegia - - - - - - | 3 |
| Lumbago - - - - - - | 4 |
| Chronic Rheumatiſm - - | 10 |
| Herpes - - - - - - - | 12 |
| Tinea - - - - - - - - | 1 |
| Papulous Eruptions - - | 16 |
| PUERPERAL DISEASES. | |
| Dolores Poſt Partum - - | 7 |
| Maſtodynia - - - - - - | 2 |
| Menorrhagia Lochialis - - | 3 |
| INFANTILE DISEASES. | |
| Diarrhœa - - - - - - | 15 |
| Herpes - - - - - - - - | 12 |
| Aphthæ - - - - - - - | 4 |

The ſeveral ſpecies of inteſtinal diſeaſe, which conſtituted ſo large a proportion of the liſt, in the report of the laſt month, ſtill continue to prevail. Children as well as adults have been much affected by them, and in many caſes they have proved very obſtinate.

